# An Open-Label Comparative Study of the Impact of Two Types of Electrical Stimulation (Direct Current Neuromuscular Electrical Stimulation and Transcutaneous Electrical Stimulation) on Physical Therapy Treatment of Diabetic Peripheral Neuropathy

**DOI:** 10.1155/jdr/9970124

**Published:** 2025-02-04

**Authors:** Dimitrios Kostopoulos, Konstantine Rizopoulos, Joe McGilvrey, Jennifer Hauskey, Jeff Courcier, Kay Connor-Israel, Harry Koster, Ramona von Leden

**Affiliations:** ^1^Department of Physical Therapy, Hands-On Diagnostics, New York, New York, USA; ^2^Department of Psychology, The University of Texas at Austin, Austin, Texas, USA; ^3^Department of Research, NeuFit, Austin, Texas, USA

**Keywords:** diabetic peripheral neuropathy, electrical stimulation, nerve conduction parameters, neuropathic symptoms

## Abstract

**Objective:**The objective of this study is to evaluate and compare the effectiveness of treatments with two different electrical stimulation (e-stim) devices—pulsed direct current (DC) (Neubie) and alternating current (AC) (transcutaneous electrical stimulation (TENS))—in the treatment of symptoms for patients with diabetic peripheral neuropathy (DPN).

**Design:**Randomized controlled trial (RCT) with parallel groups.

**Methods:** One hundred fifty participants were recruited from 13 Hands-On Diagnostics–affiliated sites across several US locations. Participants were randomly divided into two groups for comparison—Neubie and TENS. Participants received a 30-min foot stimulation protocol with either TENS unit electrodes or Neubie electrodes. Outcome measures included the Toronto Clinical Neuropathy Score (TCNS), two-point discrimination, visual analogue scale (VAS), vibration sense (VBS), nerve conduction velocity (NCV), and nerve amplitude. The effect of the two variables on all outcome measures was determined using an analysis of covariance (ANCOVA).

**Results:** The Neubie group demonstrated statistically significant improvements in TCNS for both right and left sides (*p* < 0.001), two-point discrimination of the dominant foot (*p* = 0.001), VBS (*p* = 0.022) and VAS scores (*p* = 0.009), and some but not all nerves tested by NCV (*p* < 0.05).

**Conclusion:** Overall, DPN treatment with the Neubie resulted in significant improvements in several major outcome measures, whereas TENS showed no significant difference in any outcome measure. These findings support the use of DC devices as a potentially superior therapeutic treatment for neuropathy over AC devices like the TENS unit.

**Trial Registration:** ClinicalTrials.gov identifier: NCT05442021

## 1. Introduction

Diabetic peripheral neuropathy (DPN) is a common complication in both Type 1 and 2 diabetes, affecting approximately 40%–70% of people with diabetes [1–6]. DPN results in pain, numbness, and paresthesia, most commonly in the feet and hands [7–10]. Both large and small nerve fibers are impacted by DPN, altering pain, proprioception, touch perception, and motor function, which can cause burning foot or hand pain and serve as protective mechanisms from ulcerations [11]. Traditionally, treatment for DPN has focused mainly on drug therapies, which are associated with multiple side effects, including lethargy, somnolence, and increased risk of falls [12–17]. One alternative treatment for DPN that has shown promise is the use of electrical stimulation (e-stim), as it is a noninvasive therapeutic modality that has few side effects and contraindications and no known drug interactions [18–23].

The literature on the use of e-stim for treating neuropathy symptoms demonstrates the capability of the modality to alter nerve injury or neuropathy: in diabetes, nerve damage has been associated with microvascular disease to the nerve, and cutaneous circulation significantly increases with the application of e-stim, as well as vascular endothelial growth factor (a primary angiogenic factor) [24–27]. This increase suggests that e-stim may increase angiogenesis [26, 28–32], which in turn may improve microcirculation associated with neuropathy, leading to reduced symptoms and improved nerve function [24, 25, 28, 29, 33, 34]. The application of e-stim stimulates cutaneous afferent fibers, which may contribute to the reported analgesic effect [35–46]. Additionally, preclinical studies suggest that e-stim inhibits nociception at the presynaptic level in the dorsal horn, effectively reducing pain by limiting the transmission of pain signals [43].

A limited number of clinical studies have investigated the use of e-stim for DPN symptoms. Many have used transcutaneous electrical stimulation (TENS), which employs lower frequencies and alternating currents (ACs), and have seen some effectiveness for pain associated with neuropathy but have limited impact on other symptoms [42, 43, 47–55]. In contrast, clinical studies have found that direct current (DC) neuromuscular e-stim at higher frequencies is significantly more effective than TENS at ameliorating symptoms like motor function and numbness [56–58]. In addition, the process of activating denervated muscles necessitates a longer duration of electrical pulses, which can be achieved using DC but not AC [59].

Historically, DC has been less useful in the clinical setting, as the continuous unidirectional flow of ions leads to a buildup of charge that can cause skin irritation and burns. Thus, despite the evidence supporting the effectiveness of DC over AC, most clinical applications of e-stim remain AC. Recent advances in DC technology have addressed this issue, allowing for the safe use of DC in clinical applications. One such device is the Neubie, offered by Neurological Fitness Equipment and Education LLC (NeuFit). Most commercially available DC devices address the issues of charge buildup by using very short pulse widths (5–200 ms) at high voltage. However, a longer pulse width has been found to be more effective for clinical application. To this point, the Neubie counters and eliminates the issue of irritation and pulse width with an additional carrier waveform that dissipates charge buildup, allowing for the safe use of a longer pulse width (460 ms).

Therefore, in the case of DPN, a device like the Neubie would be uniquely suited to targeting common symptoms [60, 61].

### 1.1. Hypothesis and Statement of Purpose

We hypothesized that treatment with the Neubie device would result in greater improvement in both subjective and objective symptoms related to DPN than treatment with TENS would. The results of this study could impact future recovery protocols not just for DPN but for any condition that results in nerve damage, muscle weakness, and chronic pain.

## 2. Methods

### 2.1. Selection and Description of Participants

Participants were recruited from 13 physical therapy (PT) clinics trained in EMG testing. Upon enrollment in the study, participants were assigned a number with a randomization calculator and assigned to either the experimental or control group based on the block randomization method to build two groups of equal size.

Participants were enrolled based on the following inclusion criteria:

1. Must have a minimum score of 1 (mild polyneuropathy) on the modified Toronto Clinical Neuropathy Score (TCNS).
2. Must be able to attend weekly sessions for the 6 weeks of the study.
3. Must be at least 18 years old.

Participants were excluded if they:

1. were pregnant at the time of the study.
2. had a cardiac pacemaker.
3. had active or recent cancer in the lower limbs.
4. had active or recent blood clots in the lower limbs.
5. had a history of epilepsy.
6. had open wounds.

### 2.2. Recruited Sample

One hundred fifty participants fulfilled the inclusion criteria requirements and were enlisted for the final study. All the recruited participants were above 18 years, with a group mean age of 74 years. Regarding gender, 52% were male and 48% were female, thus satisfying the desire for a gender-balanced sample. Participants obtained informed consent, and their rights were protected. Each participant was informed that data about him or her would be submitted for publication. An institutional board review was completed by Advarra.

### 2.3. Treatment Protocol

Participants underwent a specialized neuropathy protocol that included traditional PT for neuropathy and foot stimulation treatment with the Neubie or TENS unit. Subjects participated in 12 sessions of PT over 6 weeks (two per week). The sessions included a 30-min foot stimulation session with either the Neubie or TENS unit and 15 min of various standard-of-care PT exercises determined by each physical therapist. Treatments were open-label, and participants were aware of which stimulation type they received. Order of outcome measures was maintained across all sites. The treating clinician was maintained for each participant throughout the duration of the study.

• TENS group: Sessions included a 30-min TENS (50–100 Hz, pulse width of 50–200 *μ*s) application and 15 min of various PT.
• Neubie group: Neurostimulation pads were linked to electrodes designated as red or black for paired placement on the skin. Red denoted positive, black denoted negative, and the direction of current flow was from the red to the black pad.

**Figure 1:**
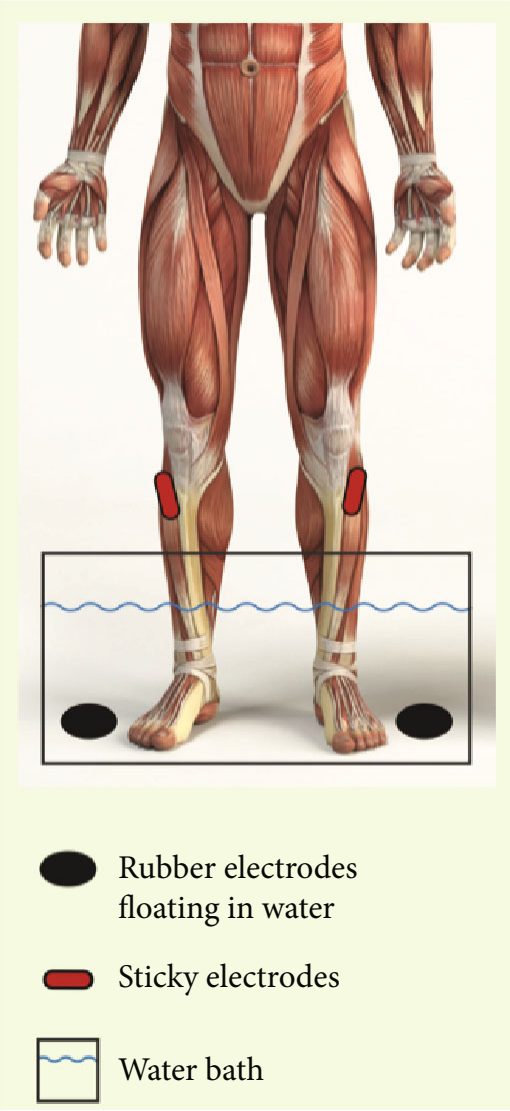
Electrical stimulation water submersion setup. Red (positive) electrodes are placed on the tibialis anterior, and black (negative) carbon fiber electrodes are placed in the water bath.

The pad placement process was standardized for uniformity. Rectangular electrodes were attached to the red leads at the tibialis anterior, and the black leads were attached to 4⁣^″^ carbon fiber electrodes and placed into a container of water. Participants' feet were placed into the water, using the water as a conductor for stimulation of the full foot and the rectangular pads to stimulate the calf (Figure 1).

e-stim frequencies used with the Neubie (460 Hz pulse width) were standardized at 500 pulses per second, as per NeuFit's protocols. Regarding stimulation intensity, participants were asked to undergo e-stim with the Neubie at their “treatment threshold.” The treatment threshold was described as “uncomfortable” but not “painful” (a 5–7 out of 10 on a perceived intensity scale). Participants were asked to mobilize their feet and ankles via joint movements in the water while being stimulated.

### 2.4. Data Collection Methods

Prior to the first session (baseline) and after the final treatment session (outcome), subjects received an evaluation that included an electrodiagnostic EMG/nerve conduction velocity (NCV) study, the TCNS assessment, pain assessment, and sensory assessment.

### 2.5. Outcome Measures

The baseline and outcome measures included TCNS for the right and left sides, two-point discrimination of the dominant foot (how far apart the patient can sense two pinpoints), VBS (vibration sense) time (how long VBS can be felt from a 128 Hz tuning fork), VAS (visual analogue scale) pain severity scores, as well as various measurements related to nerve conduction, including distal latency (DL), NCV, and amplitude (Ampl) for dominant tibial motor, dominant fibular motor, dominant ulnar motor, dominant sural, dominant superficial fibular sensory, and dominant ulnar sensory nerves.

## 3. Statistical Methods

All statistical analyses were conducted using IBM SPSS Statistics Version 27.0.1. Descriptive and inferential analyses were performed to evaluate the effectiveness of the Neubie and TENS treatments for DPN.

### 3.1. Descriptive Statistics

Sociodemographic characteristics and baseline clinical parameters were summarized using descriptive statistics. For categorical variables, frequencies and percentages were calculated, while continuous variables were summarized using means and standard deviations (SDs). The normality of the data was assessed using Q-Q plots, confirming the suitability of parametric tests.

### 3.2. Comparisons of Baseline Characteristics

To ensure comparability between the two groups, baseline characteristics were analyzed using independent samples *t*-tests for continuous variables (e.g., age and clinical parameters) and Fisher's exact test for categorical variables (e.g., gender distribution). These analyses confirmed no statistically significant differences between the groups before the intervention.

### 3.3. Adjusted Analyses

Postintervention clinical and nerve conduction parameters were analyzed using analysis of covariance (ANCOVA) to adjust for potential confounders, including age, gender, and baseline values of the respective outcome measures. This approach isolated the treatment effects by controlling for these covariates. Results from ANCOVA are reported as estimated marginal means (EMMs) with associated standard errors (SEs) and *p* values. Adjusted findings were considered statistically significant at *p* < 0.05.

### 3.4. Correction for Multiple Comparisons

To control for the increased risk of Type I error due to the analysis of multiple outcome measures, a Bonferroni correction was applied to the primary outcomes, including TCNS, two-point discrimination, VBS, VAS, and nerve conduction parameters (DL, NCV, and Ampl). This adjustment divided the alpha level (0.05) by the number of comparisons, yielding a more stringent threshold for significance. Postcorrection, only dominant ulnar motor NCV (*p* < 0.001) and dominant sural Ampl (*p* = 0.005) remained statistically significant.

### 3.5. Reporting of Results

Results are presented in three tiers:

1. Unadjusted comparisons between groups to provide an initial assessment.
2. Adjusted comparisons using ANCOVA to account for confounders.
3. Multiple comparisons–corrected results to ensure robust statistical interpretation.

### 3.6. Significance Thresholds

A two-tailed *p* value < 0.05 was considered statistically significant for all unadjusted and adjusted analyses. For Bonferroni-corrected analyses, the significance threshold was determined by dividing 0.05 by the number of comparisons, yielding a stricter criterion for statistical significance.

### 3.7. Sample Size Calculation and Power Analysis

Sample size calculation was performed using G∗Power software to ensure adequate statistical power to detect meaningful differences between the groups. The calculation targeted a moderate effect size (Cohen's *d* = 0.5) for the primary outcome measure (e.g., TCNS) with 80% power and an alpha level of 0.05. This analysis indicated a minimum requirement of 64 participants per group to achieve statistical significance. To account for potential dropouts, the recruitment target was increased to 75 participants per group, resulting in a total sample size of 150.

## 4. Results

### 4.1. Sociodemographic Characteristics

A total of 150 participants were enrolled in the study, with 75 participants in the Neubie group and 75 in the TENS group.  Table 1 presents the sociodemographic characteristics of the participants. The two groups were balanced with respect to gender distribution (*p* = 0.253) and age (*p* = 0.058). Although the Neubie group was slightly older on average (75.3 ± 5.7 vs. 72.8 ± 9.6 years for the TENS group), this difference was not statistically significant and was adjusted for in subsequent analyses.

### 4.2. Baseline Clinical and Nerve Conduction Parameters

The two groups' baseline characteristics for clinical and nerve conduction parameters were similar (Table 2). No significant differences were found in TCNS, two-point discrimination, VBS, VAS, or nerve conduction parameters such as DL, NCV, and Ampl. This similarity ensures comparability between groups before the intervention.

### 4.3. Unadjusted Postintervention Results

The unadjusted postintervention results showed that the Neubie group demonstrated statistically significant improvements in several outcome measures compared to the TENS group (Table 3).

Key findings include:

a. Significant reductions in TCNS scores for both the right and left sides (*p* < 0.001).
b. Significant improvement in two-point discrimination of the dominant foot (*p* = 0.001).
c. Enhanced VBS (*p* = 0.022).
d. Reduced pain as measured by VAS (*p* = 0.009).
e. Improvements in NCV and Ampl for several nerves, including the dominant tibial motor, fibular motor, sural, and superior fibular sensory nerves.

### 4.4. Adjusted Postintervention Results

To control for potential confounders, postintervention results were adjusted for baseline values, age, and gender using ANCOVA (Table 4). After adjustment, the Neubie group maintained significant improvements in TCNS for both the right and left sides (*p* < 0.001), two-point discrimination of the dominant foot (*p* = 0.001), and VBS (*p* = 0.022).

Significant differences were observed in NCV for the dominant fibular motor (*p* = 0.006), dominant ulnar motor (*p* < 0.001), dominant sural (*p* = 0.013), and dominant superior fibular sensory (*p* = 0.001) nerves.

Ampl improvements were significant for the dominant tibial motor (*p* = 0.019), dominant fibular motor (*p* = 0.020), dominant sural (*p* < 0.001), and dominant superior fibular sensory (*p* = 0.006) nerves.

### 4.5. Correction for Multiple Comparisons

To reduce the risk of Type I error, Bonferroni's correction was applied to the primary outcome measures. After correction, significant improvements remained for TCNS (right and left sides, *p* < 0.001), two-point discrimination (*p* = 0.001), VBS (*p* = 0.022), dominant ulnar motor NCV (*p* < 0.001), and dominant sural Ampl (*p* = 0.005).

Other observed improvements, such as VAS and certain nerve conduction parameters, did not meet the adjusted threshold for significance.

## 5. Discussion

The current study compared the therapeutic effects of Neubie and TENS treatments for patients with DPN. The Neubie group demonstrated statistically significant improvements in key clinical outcomes compared to the TENS group. This was particularly evident in TCNS scores for both right and left sides, two-point discrimination of the dominant foot, and VBS and VAS scores, indicating superior clinical outcomes related to neuropathy scores, enhanced sensory function, and reduced pain levels with Neubie treatment.

Nerve conduction studies further bolstered the findings favoring the Neubie treatment. Significant improvements were observed in nerve conduction parameters, especially in NCV and Ampl for several nerve types, suggesting that Neubie potentially optimizes nerve function in patients with DPN. Nevertheless, not all nerve conduction parameters yielded significant results, underscoring the necessity for additional exploration into facets of nerve function. Specifically, there were similar outcomes in the ulnar, fibular, and tibial nerves (showing improvements in the Neubie group for DL and NCV, but not in Ampl). This suggests a more specific effect of the Neubie that encourages increased speed of signaling, but not the Ampl.

Further studies should investigate if this effect is consistent with other nerves or if the application of the e-stim is given in a different protocol. One explanation for these results could be the placement of the red electrode on the tibialis anterior. Previous studies using the Neubie have shown that the stimulation of the current from the Neubie is directly related to pad placement. The pads' placement will encourage more specific muscle activation in those regions [62]. The placement used in this study would more specifically activate the nerve fibers running through that leg region.

A possible explanation for the improved outcomes in the Neubie group over the TENS group is the differences in frequency and wavelength between the two e-stim types. Since it is DC, the Neubie operates with different frequency and wavelength ranges than conventional TENS units. Previous clinical studies have found that DC neuromuscular e-stim at higher frequencies is significantly more effective than TENS at ameliorating symptoms like motor function and numbness [56–58]. The results of the current study support these previous findings, showing a more effective treatment outcome with DC for ameliorating DPN symptoms. The changes to nerve function suggest that the longer pulse durations employed by the Neubie are sufficient to activate denervated muscles and support new nerve activity [59].

Further, it is postulated that varying frequencies and wavelengths can have distinct effects on nerve activation, repair, and overall function. The Neubie might modulate nerve activity to promote faster repair or better nerve function.

It is interesting to note that in this study, we only applied current in one direction. It would be useful to investigate alternating the polarity of the current to determine if that plays a role in the impact the current has on specific nerve signaling outcomes.

Compelling research done in spinal cord injury has shown that nerve regeneration enhanced by DC e-stim tends to grow towards one electrode, or rather with the direction that it is stimulated. This may contribute to a possible explanation as to why the improvements we see in the nerves are not consistent across all nerves stimulated [59]. Future research could include a protocol with alternating polarity to determine the overall impact on improved nerve outcomes.

## 6. Conclusion

The primary objective of this research was to discern any distinguishable therapeutic advantages of the Neubie as opposed to the conventional TENS unit for patients with DPN. Through comprehensive statistical analyses and subsequent evaluation, Neubie has demonstrated superior outcomes in several vital clinical and nerve conduction metrics. This supports our hypothesis that treatment with the Neubie device results in an improvement in both subjective and objective symptoms related to DPN.

### 6.1. Study Limitations

While the above points offer plausible reasons for the observed superior outcomes with Neubie, it is vital to note that not all nerve conduction parameters demonstrated significant results. Additionally, long-term outcomes and the sustainability of improvements need further exploration. Although the objective measurements of nerve function were significantly improved at the end of treatment, it is unknown how long these improvements would be seen after cessation of treatment. Follow-up measurements within the weeks to months following would greatly improve our understanding of the ability of electrotherapy to create a lasting change in nerve function.

When examining subjective outcome measures like reported pain or sensory inputs, the potential placebo effect or the heightened expectation of results with newer technologies must be considered. Future studies might incorporate a double-blinded design to more effectively control these effects.

In summary, Neubie has shown promising results over TENS in this study for the treatment of DPN, but a holistic understanding of its efficacy would require more extensive studies, long-term follow-ups, and further exploration of mechanistic insights into its therapeutic advantages.

## 7. Key Points

### 7.1. Findings

DPN treatment with the Neubie resulted in significant patient improvements compared to TENS.

### 7.2. Implication

Future recovery protocols for DPN and conditions that result in nerve damage, muscle weakness, and chronic pain should consider recommending the Neubie device.

## Figures and Tables

**Table 1:** Comparison of sociodemographic characteristics of participants in the control and experimental group.

	**Group**												
	**Control group**				**Experimental group**				**Total**				**p** ** value**
	**N**	**%**	**M**	**SD**	**N**	**%**	**M**	**SD**	**N**	**%**	**M**	**SD**	
Gender													
Female	40	53.3%			32	42.7%			72	48.0%			0.253^a^
Male	35	46.7%			43	57.3%			78	52.0%			
Total	75	100%			75	100%			150	100%			
Age			72.8	9.6			75.3	5.7			74.1	8.0	0.058^b^

^a^Fisher's exact test.

^b^Independent samples *t*-test.

**Table 2:** Comparison of baseline clinical/nerve conduction parameters of participants in the experimental and control group.

	**Group**						
	**Control group**		**Experimental group**		**p** ** value** ^**a**^	**Total**	
	**M**	**SD**	**M**	**SD**		**M**	**SD**
TCNS right pre	11.4	3.1	11.6	2.7	0.560	11.5	2.9
TCNS left pre	11.3	3.0	11.5	2.8	0.716	11.4	2.9
Two-point discrimination dominant foot pre (mm)	33.7	7.6	32.8	6.9	0.481	33.3	7.3
VBS (vibration sense) time pre	2.7	1.6	2.7	1.1	1.000	2.7	1.4
VAS (visual analogue scale) pre	5.1	1.8	5.1	1.1	1.000	5.1	1.5
Dominant tibial motor							
DL pre (msec)	4.5	0.8	4.3	0.8	0.142	4.4	0.8
NCV pre (m/sec)	38.7	4.9	38.2	5.1	0.581	38.5	5.0
Ampl pre	4.4	2.7	4.3	3.1	0.951	4.4	2.9
Dominant fibular motor							
DL pre (msec)	4.9	1.0	4.9	1.0	0.724	4.9	1.0
NCV pre (m/sec)	39.4	4.8	38.7	5.3	0.403	39.1	5.1
Ampl pre	1.8	1.3	2.0	1.4	0.600	1.9	1.3
Dominant ulnar motor							
DL pre (msec)	3.1	0.6	3.1	0.5	0.701	3.1	0.6
NCV pre (m/sec)	51.1	8.6	51.0	7.8	0.953	51.0	8.2
Ampl pre	7.3	1.8	7.6	2.0	0.273	7.5	1.9
Dominant sural							
DL pre (msec)	2.8	2.0	3.0	2.1	0.468	2.9	2.1
NCV pre (m/sec)	25.6	19.0	24.3	17.4	0.670	25.0	18.2
Ampl pre	2.0	1.9	2.1	2.0	0.790	2.1	1.9
Dominant sup fibular sensory							
DL pre (msec)	4.1	3.2	4.7	3.3	0.260	4.4	3.3
NCV pre (m/sec)	18.6	15.5	16.9	12.9	0.474	17.8	14.3
Ampl pre	1.4	1.5	1.5	1.5	0.806	1.5	1.5
Dominant ulnar sensory							
DL pre (msec)	2.9	1.7	3.1	1.6	0.477	3.0	1.6
NCV pre (m/sec)	31.0	17.6	33.1	16.4	0.443	32.1	17.0
Ampl pre	7.8	5.6	8.4	5.3	0.466	8.1	5.5

^a^Independent samples *t*-test.

**Table 3:** Comparison of postintervention clinical/nerve conduction parameters of experimental and control group (not adjusted for confounders).

	**Group**						
	**Control group**		**Experimental group**		**p** ** value**⁣^∗^	**Total**	
	**M**	**SD**	**M**	**SD**		**M**	**SD**
TCNS right pre	10.9	3.4	10.1	2.7	0.118	10.5	3.1
TCSN left pre	10.6	3.2	10.0	2.6	0.245	10.3	2.9
Two-point discrimination dominant foot pre (mm)	33.0	7.6	31.0	7.1	0.105	32.0	7.4
VBS (vibration sense) time pre	2.9	1.6	3.3	1.4	0.149	3.1	1.5
VAS (visual analogue scale) pre	4.2	2.1	3.8	1.1	0.138	4.0	1.6
Dominant tibial motor							
DL post (msec)	4.4	0.9	4.3	0.8	0.262	4.3	.8
NCV post (m/sec)	39.0	5.0	40.6	7.5	0.139	39.8	6.4
Ampl post	4.4	2.7	4.7	3.0	0.478	4.6	2.9
Dominant fibular motor							
DL post (msec)	4.8	0.9	4.7	0.9	0.451	4.8	0.9
NCV post (m/sec)	40.1	5.2	41.6	5.0	0.087	40.9	5.1
Ampl post	1.9	1.2	2.3	1.4	0.140	2.1	1.3
Dominant ulnar motor							
DL post (msec)	3.1	0.5	3.1	0.5	1.000	3.1	0.5
NCV post (m/sec)	51.7	8.0	54.2	7.5	0.048⁣^∗^	52.9	7.8
Ampl post	7.2	1.6	7.7	1.9	0.100	7.5	1.8
Dominant sural							
DL post (msec)	2.7	2.0	2.7	1.9	0.973	2.7	1.9
NCV post (m/sec)	26.2	19.2	26.7	18.3	0.886	26.4	18.7
Ampl post	2.1	1.9	3.1	2.4	0.005⁣^∗^	2.6	2.2
Dominant sup fibular sensory							
DL post (msec)	4.1	3.1	4.3	3.0	0.680	4.2	3.1
NCV post (m/sec)	18.8	15.7	18.8	14.3	0.994	18.8	14.9
Ampl post	1.5	1.5	1.7	1.6	0.351	1.6	1.6
Dominant ulnar sensory							
DL post (msec)	2.9	1.8	3.0	1.6	0.907	2.9	1.7
NCV post (m/sec)	30.1	17.6	35.3	17.6	0.072	32.7	17.7
Ampl post	8.3	8.1	9.7	6.0	0.238	9.0	7.1

⁣^∗^Independent samples *t*-test.

**Table 4:** Comparison of postintervention clinical/nerve conduction parameters of experimental and control group participants (adjusted for age, gender, and respective baseline parameter).

	**Group**				
	**Control group**		**Experimental group**		**p** ** value**⁣^∗^
	**EEM**	**SE**	**EEM**	**SE**	
TCNS right post	11.06	0.14	9.99	0.14	< 0.001⁣^∗^
TCSN left post	10.71	0.14	9.91	0.14	< 0.001⁣^∗^
Two-point discrimination dominant foot post (mm)	32.54	0.24	31.38	0.25	0.001⁣^∗^
VBS (vibration sense) time post	2.909	0.11	3.28	0.11	0.022⁣^∗^
VAS (visual analogue scale) post	4.21	0.13	3.70	0.13	0.009⁣^∗^
Dominant tibial motor					
DL post (msec)	4.38	0.08	4.29	0.08	0.461
NCV post (m/sec)	38.97	0.74	40.78	0.75	0.088
Ampl post	4.34	0.15	4.84	0.148	0.019⁣^∗^
Dominant fibular motor					
DL post (msec)	4.81	0.08	4.72	0.08	0.397
NCV post (m/sec)	39.83	0.48	41.77	0.484	0.006⁣^∗^
Ampl post	1.99	0.06	2.20	0.064	0.020⁣^∗^
Dominant ulnar motor					
DL post (msec)	3.08	0.04	3.10	0.04	0.771
NCV post (m/sec)	51.60	0.49	54.41	0.496	< 0.001⁣^∗^
Ampl post	7.342	0.11	7.58	0.11	0.112
Dominant sural					
DL post (msec)	2.81	0.05	2.61	0.05	0.003⁣^∗^
NCV post (m/sec)	25.58	0.50	27.40	0.51	0.013⁣^∗^
Ampl post	2.13	0.15	3.14	0.148	< 0.001⁣^∗^
Dominant sup fibular sensory					
DL post (msec)	4.34	0.08	3.97	0.08	< 0.001⁣^∗^
NCV post (m/sec)	17.90	0.39	19.70	0.39	0.001⁣^∗^
Ampl post	1.53	0.04	1.69	0.04	0.006⁣^∗^
Dominant ulnar sensory					
DL post (msec)	3.02	0.08	2.88	0.08	0.245
NCV post (m/sec)	30.94	0.75	34.36	0.76	0.002⁣^∗^
Ampl post	8.58	0.68	9.39	0.68	0.405

⁣^∗^Analysis of covariance (ANCOVA) (fixed effects: experimental/control group and gender; covariates: age and respective preintervention parameter).

## Data Availability

The data that support the findings of this study are available on request from the corresponding author, which must include a description of which data are available, from whom the data are available, how they should be contacted, and how data may be reused. The data are not publicly available due to privacy or ethical restrictions (approved by Advarra IRB, Protocol # Pro00063515).
